# A Cross-Sectional Study on Cerebral Hemodynamics After Mild Traumatic Brain Injury in a Pediatric Population

**DOI:** 10.3389/fneur.2018.00200

**Published:** 2018-04-05

**Authors:** Corey M. Thibeault, Samuel Thorpe, Michael J. O’Brien, Nicolas Canac, Mina Ranjbaran, Ilyas Patanam, Artin Sarraf, James LeVangie, Fabien Scalzo, Seth J. Wilk, Ramon Diaz-Arrastia, Robert B. Hamilton

**Affiliations:** ^1^Neural Analytics, Inc., Los Angeles, CA, United States; ^2^Citadel LLC, Greenwich, CT, United States; ^3^Departments of Neurology and Computer Science, University of California, Los Angeles, CA, United States; ^4^Penn Presbyterian Medical Center, University of Pennsylvania Hospital, Philadelphia, PA, United States

**Keywords:** traumatic brain injury, vascular reactivity, cerebral blood flow autoregulation, blood flow, cerebral blood flow velocity

## Abstract

The microvasculature is prominently affected by traumatic brain injury (TBI), including mild TBI (concussion). Assessment of cerebral hemodynamics shows promise as biomarkers of TBI, and may help inform development of therapies aimed at promoting neurologic recovery. The objective of this study was to assess the evolution in cerebral hemodynamics observable with transcranial Doppler (TCD) ultrasound in subjects suffering from a concussion at different intervals during recovery. Pediatric subjects between the ages of 14 and 19 years clinically diagnosed with a concussion were observed at different points post-injury. Blood flow velocity in the middle cerebral artery was measured with TCD. After a baseline period, subjects participated in four breath holding challenges. Pulsatility index (PI), resistivity index (RI), the ratio of the first two pulse peaks (P2R), and the mean velocity (MV) were computed from the baseline section. The breath hold index (BHI) was computed from the challenge sections. TCD detected two phases of hemodynamic changes after concussion. Within the first 48 h, PI, RI, and P2R show a significant difference from the controls (*U* = −3.10; *P* < 0.01, *U* = −2.86; *P* < 0.01, and *U* = 2.62; *P* < 0.01, respectively). In addition, PI and P2R were not correlated (*r*_p_ = −0.36; *P* = 0.23). After 48 h, differences in pulsatile features were no longer observable. However, BHI was significantly increased when grouped as 2–3, 4–5, and 6–7 days post-injury (*U* = 2.72; *P* < 0.01, *U* = 2.46; *P* = 0.014, and *U* = 2.38; *P* = 0.018, respectively). To our knowledge, this is the first longitudinal study of concussions using TCD. In addition, these results are the first to suggest the multiple hemodynamic changes after a concussion are observable with TCD and could ultimately lead to a better understanding of the underlying pathophysiology. In addition, the different hemodynamic responses to a concussion as compared to severe traumatic brain injuries highlight the need for specific diagnostic and therapeutic treatments of mild head injuries in adolescents.

## Introduction

Alterations in vascular function are one of the many pathologies accompanying the structural and metabolic changes observed after a traumatic brain injury (TBI) (1). These abnormalities in cerebral blood flow (CBF) have been found in moderate to severe traumatic brain injuries (1–5) as well as in mild traumatic brain injuries (mTBI, also referred to as concussions) (6–13). The mechanism for these changes is still not fully established, but it is clear that both the initial insult and the secondary injury response contribute to the observed vascular pathology (1).

Over the first days to weeks after injury, prior research has identified a distinct progression of vascular dysfunction. In severe TBI, phases have been described that begin with hypoperfusion, evolve into hyperemia, and finally end in a period of vasospasm (2, 4). Similarly, for adults suffering a concussion, there is a clear vascular dysfunction, with a number of studies showing alterations in CBF, but the recovery progression does not appear to match what is observed in severe TBI. Children with mTBI, however, exhibit reactivity impairments similar to moderate or severe TBI, with an initial period of increased CBF followed by a relative decrease compared to baseline (14). Age clearly matters in CBF regulation in both initial risk and during recovery (15). A better understanding of the pediatric brain after suffering a concussion and during recovery is vital to improving diagnostic, prognostic, and therapeutic tools. Methods for monitoring and understanding how the vasculature changes are one step toward improving that. In addition, there is an immediate need for more quantitative, physiologically based measures for concussion management.

Transcranial Doppler (TCD) ultrasound provides a non-invasive method of monitoring cerebral blood flow velocity (CBFV). Although not a direct measure of CBF, this signal provides insight into vascular dysfunction. In mTBI cases, standard neuroimaging studies display no abnormalities (8). TCD, however, has captured alterations in vascular function in a few key studies. Len et al. (6) showed differences in cerebrovascular reactivity (CVR) during a hypocapnia challenge in a population who had recently suffered a concussion. In a subsequent study, they then showed that TCD could also capture CVR abnormalities under hypercapnia (16). Similarly, Bailey et al. (9) showed lowered CVR in a population suffering from chronic mTBI. There has not however, been a study using TCD that captures the progression of hemodynamic dysfunction after a concussion through the acute stage (<48-h post-injury).

The purpose of this study was to assess the evolution in cerebral hemodynamics observable with TCD in subjects suffering from a concussion. Blood flow velocity in the middle cerebral artery was measured and CVR was evaluated with breath holding challenges. During the different phases of the exam, traditional TCD features, Gosling pulsatility index (PI), resistivity index (RI), the ratio of the first two pulse peaks (P2R), and the mean velocity (MV) were extracted and the breath hold index (BHI) was computed from the challenge sections to estimate CVR. These features showed significant differences at the population level that were dependent on how far from the injury the subjects were scanned. This study suggests that there are different phases of hemodynamic dysfunction and that the progression of those phases is different in pediatric patients as compared to that seen in adults. The use of relatively inexpensive non-invasive methods is promising as a biomarker of vascular injury and recovery after mTBI.

## Materials and Methods

### Participants

**Figure 1:**
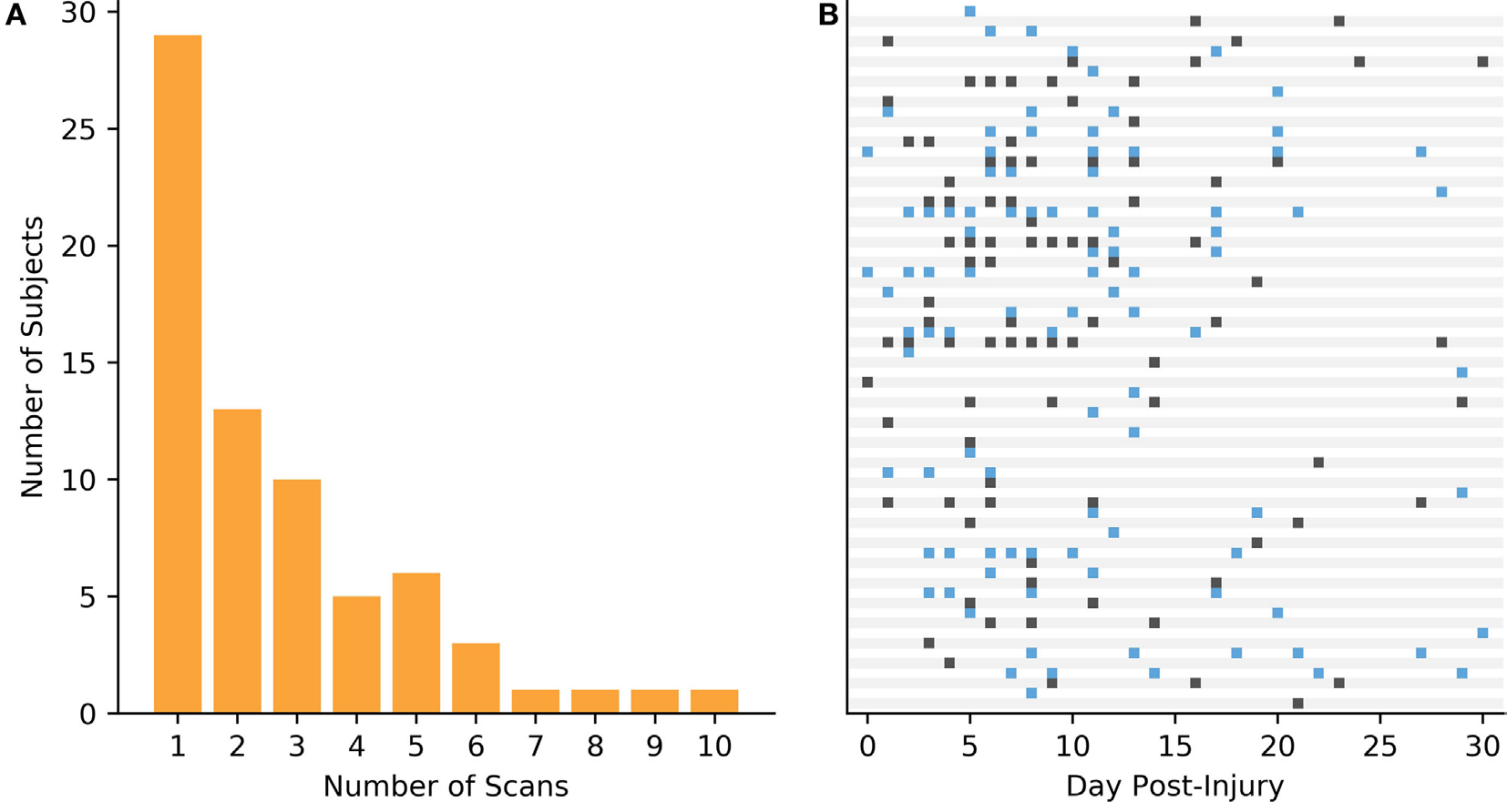
Data collection for the mild traumatic brain injury (mTBI) subjects. **(A)** Breakdown of the frequency that the subject population was scanned. **(B)** Scan times in days post-injury for each of the mTBI cases.

A total of 179 subjects between the ages of 14 and 19 years old were recruited from participating clinics and high schools in Los Angeles, CA, USA. Seventy Subjects with concussions clinically diagnosed by independent practicing physicians blinded to the TCD results over a range of 1 h to 30 days post-injury were selected. There were a total of 187 scans collected for the case group (labeled mTBI), for a median of 2 scans per subject. Note that multiple scans for a single patient were collected at different days post-injury. The overall scan frequency is illustrated in Figure 1. The control group consisted of 109 age- and activity-matched subjects, who were scanned only once. All data collection and processing was approved by Western Institutional Review Board (IRB #20141111).

### Data Collection

Transcranial Doppler (TCD) signals were collected with the DWL Doppler Box-X (DWL USA Inc.), and 2 MHz ultrasound probes held in place by a thermally molded bracket attached to an adjustable headband strap. The middle cerebral arteries were then insonated transtemporally by trained ultrasound technicians. To ensure compliance with the breath holding protocol the end-tidal CO_2_ was collected through a nasal cannula using the Nonin Medical RespSense capnometer. The data streams, patient history, and clinical measures were collected with a custom codebase running in the Windows 8/10 operating systems.

### Protocol

**Figure 2:**
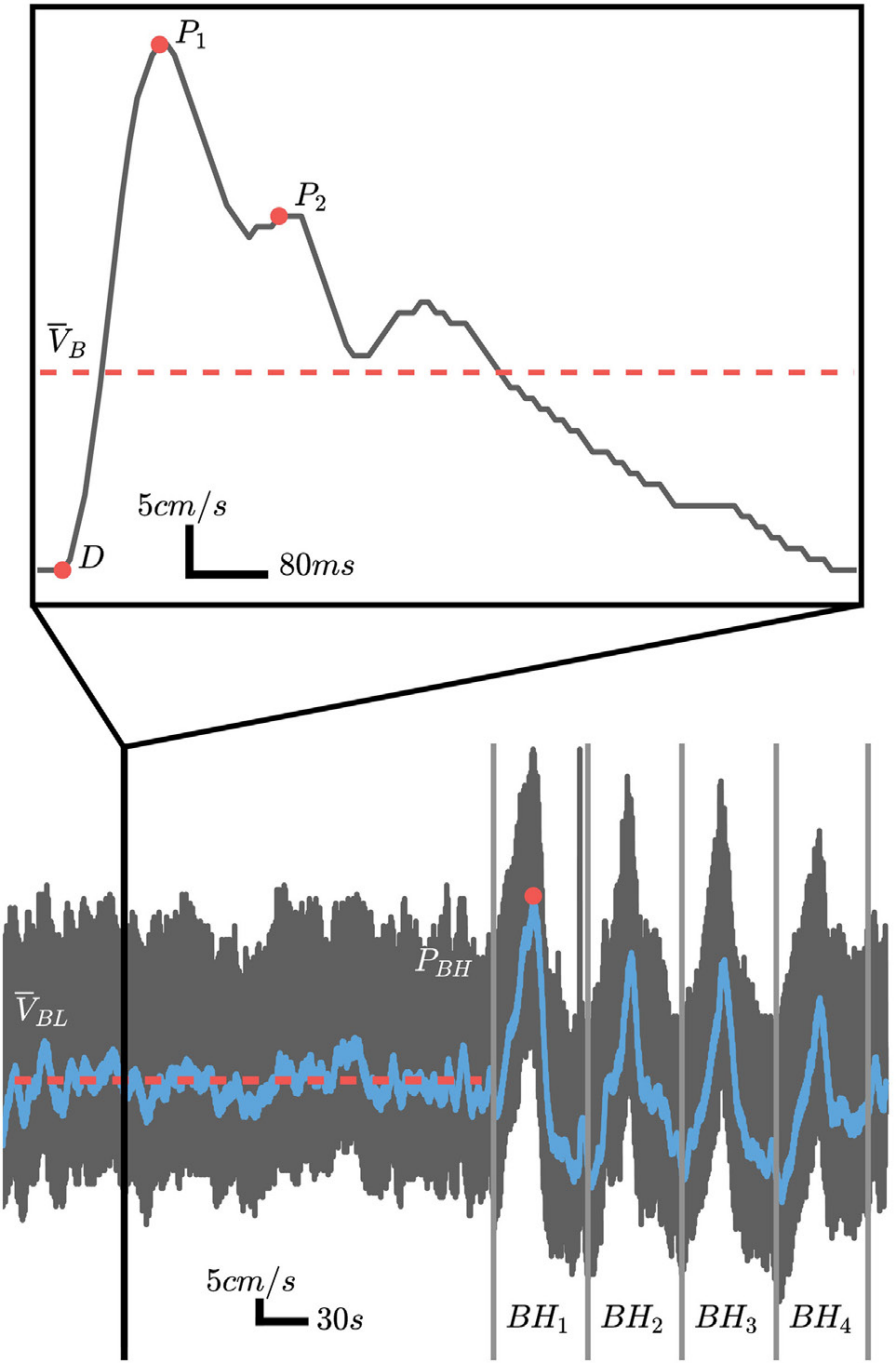
Representative cerebral blood flow velocity (CBFV) signal over the course of the experimental protocol (black trace) with the filtered signal used for cerebrovascular reactivity (CVR) calculations overlaid (blue trace). For CVR calculations, the baseline mean velocity (MVb) is computed over the period highlighted by the solid red line and the peak is velocity is computed from the breath hold period with the highest peak as illustrated by the red circle in BH1. The pulsatile analysis is computed using baseline pulses as illustrated by the inlay outlined by the solid vertical black lines. The systolic (P1), diastolic (D), and second peak (P2) are marked by the solid red circles.

The data collection consisted of several sections illustrated in Figure 2. During the initial 5-min baseline section, subjects were instructed to breath normally through their nose. The subjects were then instructed to take a normal breath and hold it. Breath holding lasted for 25 s and was followed by a 35-s recovery period. Research into the optimal duration for the breath hold is limited; however, previous work has shown that 20- to 40-s durations are sufficient to provide a CBFV response (17, 18). The breath hold was repeated four times. Using normal inspiration can help limit the Valsalva effect which would cause an initial decrease in mean CBFV and lead to underestimation of reactivity (18).

### Data Analysis—Pulsatile Features

The pulsatile features were extracted from the initial 5-min baseline period. Individual beats were identified automatically using proprietary software developed in Python. Four traditional features were extracted from the morphological points (illustrated in Figure 2) for each of the pulses as described below.

Pulsatility index is often considered a measure of cerebrovascular resistance. However, it is actually a complex metric that is influenced by the combinations of cerebral perfusion pressure, cerebrovascular resistance, arterial bed compliance, heart rate, and the pulse amplitude (19). It is calculated by

$$PI=\left(P_{1}-D\right)/\bar{V}$$

where *P*_1_ is the systolic peak, *D* is the diastolic valley, and $\bar{V}$ is the MV of the pulse. Similarly, the RI is simply the average of the diastolic and systolic values. It is related to PI but removes the effect that heart rate and waveform shape may have. This can be computed by

$$RI=\left(P_{1}-D\right)/\left[\left(P_{1}+D\right)/2\right]$$

The feature P2R is a metric for relating the second observed waveform peak, P_2_, with the systolic peak P_1_. This has been hypothesized to be related to distal bed compliance dynamics but there is currently no clear physiological link to changes in P2R. It is computed using

$$P2R=P_{2}/P_{1}$$

The MV, ${\bar{V}}_{B}$, is the average of the individual beats velocities found using

$${\bar{V}}_{B}=\frac{1}{N}\sum_{n=0}^{N}{\bar{V}}_{n}$$

where *N* is the number of beats identified and ${\bar{V}}_{n}$ is the MV for pulse *n*.

### Data Analysis—Reactivity Analysis

To evaluate the CVR, the BHI was computed by extracting the DC component of the velocity signal. This was accomplished by first low-pass filtering the global signal and then picking the highest peak between the four breath hold regions of the exam (Figure 2). The MV over the entire baseline, ${\bar{V}}_{BL}$, is computed and the BHI can be found from

$$BHI=\frac{P_{BH}-{\bar{V}}_{BL}}{{\bar{V}}_{BL}}$$

### Statistical Analysis of TCD Data

The statistical analysis was performed in Python using the SciPy library (20). The measurements between cases and controls were compared using the Wilcoxon–Mann–Whitney two-sample rank-sum test. The effect size is reported using the common language effect size statistic CL, which is the proportion of measurement pairs between cases and controls that support the hypothesis. In addition, the differences between measurements were estimated using the Hodges–Lehmann estimator (Δ) for the median difference between the populations.

## Results

### Subject History

Table 1 presents the demographic and activity breakdown for the two populations. There was no significant difference in ages between the groups (see Table 2). However, there was a difference in the total number of self-reported head hits in the past [control: 1.03 (1.32), mTBI: 2.11 (3.50), see Table 2]. There was also a higher incidence of headache leading to missed activities reported by the mTBI population —56 cases compared to 16 in the control group.

**Table 1:** Subject demographic and sport information.

	Control	Mild traumatic brain injuries (mTBI)
**Demographic information**		
Number of exams	109	187
Number of subjects	109	70
Males	97. 89%	45. 64%
Mean age (SD) years	16.06 (1.56)	16.21 (1.16)
Past head hits	1.03 (1.32)	2.11 (3.50)
**Sport breakdown**		
Football	77	31
Soccer	8	12
Other	24	27

**Table 2:** Statistical analysis of the presented results.

Comparison	*U*	*n*_c_	*n*_mTBI_	*P*	Δ	CL
Population age	0.18	70	109	0.86	–	–
Self-reported past head hits	3.51	69	64	<0.01	1.00	0.57
Pulsatility index, first 48 h	−3.10	109	11	<0.01	−0.113	0.78
Resistivity index, first 48 h	−2.86	109	11	<0.01	−0.042	0.76
P2R, first 48 h	2.62	109	11	<0.01	0.071	0.74
Breath hold index (BHI), first 48 h	−0.42	109	11	0.67	−1.79	0.46
BHI, 2–3 days post-injury	2.72	109	17	<0.01	13.56	0.71
BHI, 4–5 days post-injury	2.46	109	23	0.014	6.54	0.66
BHI, 6–7 days post-injury	2.38	109	27	0.018	5.85	0.65
BHI, 8–9 days post-injury	1.83	109	23	0.067	4.59	0.62
End-tidal CO_2_ after breath hold	−0.648	174	105	0.517	−0.007	0.52

There were a total of 14 mTBI subjects who reported having experienced loss-of-consciousness due to injury in the past, compared to only two control subjects. In addition, six of the mTBI subjects reported being knocked-out in the 12 months preceding the scan. Of those, one subject reported having to be hospitalized, four lost consciousness for less than a minute, and one subject chose to not provide a response. There was no history of loss-of-consciousness within the previous 12 months reported in the control population.

For the mechanism of injury in the mTBI population, 60 reported being injured while playing sports, 2 were injured in a car accident, 2 were injured in a fall, 1 reported other, and 5 did not provide a response.

Of the 70 case subjects, 41 had multiple scans, with a median of 2.0 and IQR of 2.75 scans. Thirty-four of those had less than 6 total scans. During analysis, the different scans for the mTBI population were grouped based on when the scan was collected (Table 3). Note that the first group, 0-1, encapsulates the first 48 h.

**Table 3:** Exam statistics.

Group (days post-injury)	Number of exams	Mean (SD) (days)
0–1	11	0.73 (0.45)
2–3	17	2.65 (0.48)
4–5	23	4.57 (0.50)
6–7	27	6.41 (0.49)
8–9	23	8.35 (0.48)
10–11	22	10.68 (0.47)
12–13	16	12.62 (0.48)
14–18	20	16.30 (1.31)
19–30	28	23.86 (3.93)

### Baseline Pulsatile Analysis

**Figure 3:**
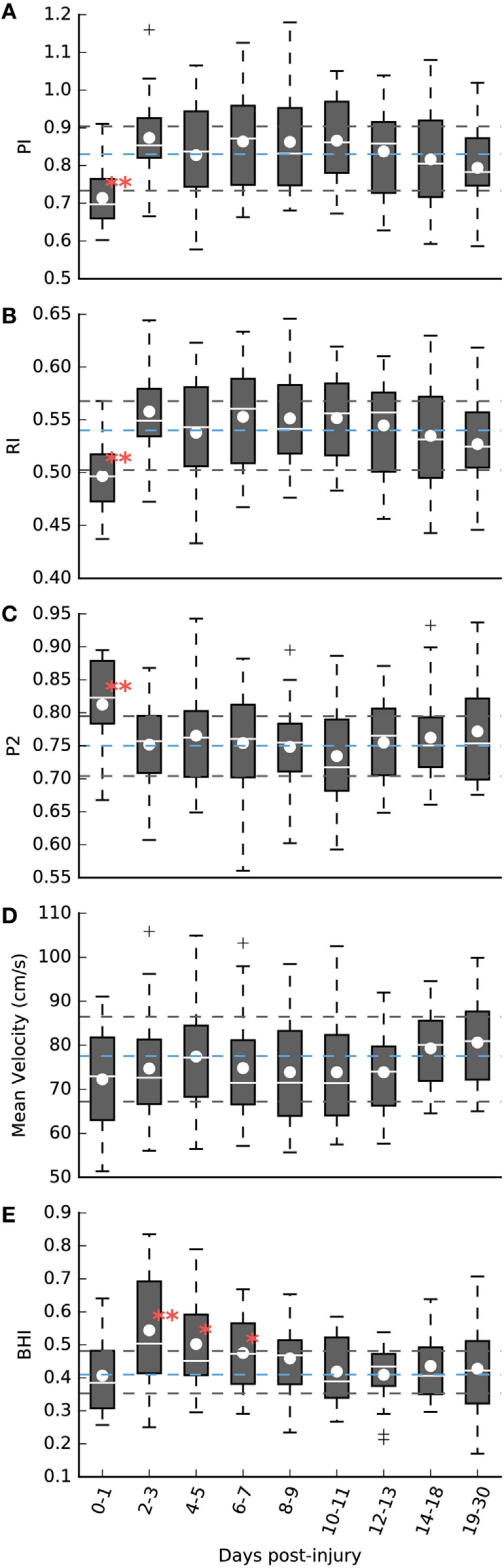
**(A–D)** Pulsatile analysis of the baseline exam period over different days post-injury. **(A)** Pulsatility index (PI). **(B)** Resistivity index (RI). **(C)** P2 ratio. **(D)** Mean velocity (MV, cm/s). **(E)** Cerebrovascular reactivity (CVR) analysis—breath hold index (BHI). **P < 0.01, *P < 0.05. The dashed blue lines represent the control population mean with interquartile range marked by the surrounding dashed black lines.

As illustrated in Figure 3A, the PI of the mTBI population was significantly lower during the first 48 h after injury (see Table 2). After that period, there was no statistical difference between cases and controls. Similarly, the RI was also significantly lower only during that initial period (see Table 2; Figure 3B). Conversely, the P2R ratio was significantly higher during the first 48 h (see Table 2; Figure 3C). The MV of the mTBI population did not differ significantly from the control population throughout the injury recovery period measured here (see Table 2; Figure 3D).

**Figure 4:**
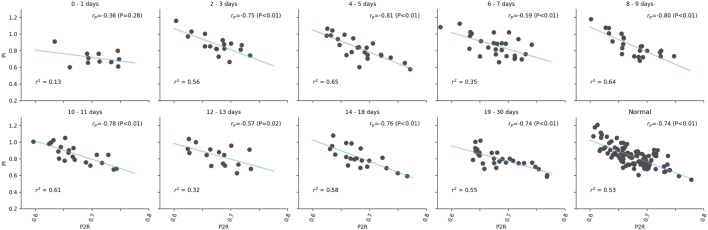
Correlation analysis between pulsatility index (PI) and P2R over different days post-injury. The linear fit, blue line, and *r*^2^ values are given for each set of values. Pearson’s *r* values are presented with corresponding *P* values in the upper right of each subplot.

While both PI and P2R of the mTBI population showed differences from the control population in the first 48 h, they are uncorrelated from each other based on Pearson’s correlation analysis (*r*_p_ = −0.36; *P* = 0.23), as illustrated in Figure 4. However, after the initial period, there was a strong negative correlation matching that observed in the controls (*r*_p_ = −0.74; *P* < 0.01). The initial lack of correlation was also observed between RI and P2R (initial 48 h: *r*_p_ = −0.14; *P* = 0.69, Controls: *r*_p_ = −0.64; *P* < 0.01). This was not the case between PI and RI (initial 48 h: *r*_p_ = 0.99; *P* < 0.01, Controls: *r*_p_ = 0.98; *P* < 0.01).

### Reactivity Analysis

The reactivity analysis, as illustrated in Figure 3E, resulted in a different period of significance compared to the pulsatile analysis. During the initial 48-h period, the BHI of the mTBI population was not different from the control population (see Table 2). After 48 h, BHI increased significantly over the control population peaking between days 2–3 after injury (see Table 2). This increase remained significant, although decaying, over days 4–5 (see Table 2), and days 6–7 (see Table 2). Over the period of days 8–9, a small difference is observed, but is no longer significant when compared to the control population (see Table 2). For the remaining post-injury periods, there was no observable difference. There was no statistical difference between the mean percent change in end-tidal CO_2_ after breath holding [Control: 12.05% (9.64%), mTBI: 12.97% (9.56%), see Table 2]. Similarly, there was no difference when comparing the cases grouped by days after injury.

## Discussion

Initial symptoms following a concussion are generally attributed to microstructural changes in neural tissue (7). Previous studies have shown a decrease in CBF during this period (8, 13, 21). Although this was not observed in the population examined here, it appears that the mechanical insult was still captured by TCD through an analysis of the pulse level features. Traditionally, PI has been attributed to cerebrovascular resistance (22, 23). However, recently a more complex relationship between PI and the remote vasculature has been proposed (19). Similarly, P2R has been shown to be correlated with intracranial pressure (24). One theory is that this is related to the compliance of the resistance vessels (25). We hypothesize that both PI and P2R are related to the distal vasculature (arterioles), the more likely area to be affected during an external biomechanical force. In this study, PI decreased while P2R increased significantly, within the population. This runs counter to what has been observed in more severe injuries, where raised PI has been used an indicator of high intracranial pressure (26), as well as a predictor of secondary neurological deficit (26, 27). The extent of the force, or severity of injury, appears to modulate the corresponding effect on PI and P2R.

Although the mean CBFV values measured here were not statistically significant between populations, there appears to be a trend of lower CBFV the closer the measurement was taken to the injury. Given that reduced CBF is a hallmark of moderate and severe TBIs, it is tempting to attribute the increased BHI values to a lower starting CBFV but a similar peak. However, the lowest velocities were observed within the first 48 h of injury, a point where the BHI values did not differ from the normal population. In addition, the recovery periods that did have significantly higher BHIs did not have CBFV values that differed statistically or visually, compared to the normal population. The lack of a significant decrease in CBFV does not indicate that CBF was unaltered in this study, only that that change was not captured.

An intriguing aspect of these results was that both PI and the P2R were significantly different at the population level but were uncorrelated. This may suggest that these are capturing different physiological mechanisms of injury. It is not clear at this time exactly which injury manifestations are affecting the pulse morphology metrics and additional research is needed to help elucidate possible mechanisms. It is important to note that these differences in waveform features are significant at the population level. As more information is collected, the heterogeneity in both individuals and injuries will likely reveal subtle differences in the progression of these features over the course of recovery.

The reactivity analysis appears to reveal the second phase of injury progression that is related to the well-established neurometabolic cascade. The surprising deviation from severe TBI was the delayed higher levels of CVR for the concussed cases. Len et al. (16) demonstrated a similar increase in CVR using TCD and breath holding for 20 subjects. However, that increase was only significant on day 2 post-injury and there were no prior measurements recorded. A similar result was shown in a pilot study with seven subjects using MRI by Militana et al. (10). Like this study, both failed to capture any significant changes in mean CBFV or CBF. In addition, the small CVR study utilizing MRI from Mutch et al. (28), showed that the two acute stage subjects, measured at days 7 and 13 post-injury, had an increase in CVR compared to normal subjects. However, those subjects were more severely injured than those measured in this study.

The hyperreactivity may be attributable to a number of metabolic and ionic responses that occur after a concussive injury. One compelling explanation previously proposed by Militana et al. (10) is an increase in nitric oxide synthase (NOS) in the endothelium. Although a number of TBI experimental paradigms have demonstrated that effect, the increase did not last beyond 48 h post-injury (29–31). After this period, NOS is significantly decreased in severe TBI—where the vascular smooth muscle remains responsive to nitric oxide but the NOS produced by endothelial cells is insufficient to elicit an appropriate response (1). This has prompted the use of PDE5 inhibitors, such as sildenafil, in the treatment of severe TBIs (1). Our results here suggest that such a treatment in the case of pediatric concussion may not be appropriate. The complexity of the neurovascular unit and injury recovery illustrate the need for more quantitative measures of the cerebral vasculature in aiding clinicians in treating patients at all levels of TBI severity.

One limitation of this study is the relatively small number of case in the first 24 h. Although the large effect size supports the conclusions and suggests that this was not a limiting factor, more measurements during the hyperacute stages are needed. In addition, these results are for the population as a whole, not for individual subjects. A follow-up study looking at individual cases over time is being conducted to determine if these results are found at the individual subject level. Another limitation was the age range of the subjects. A pediatric population was the focus here, but CBFV is known to decrease with age (32). In addition, as discussed above, adults respond differently to a TBI. Future work will expand the age range of the subjects in an effort to capture these differences. The use of breath holding can also introduce discrepancies in the amount of CO_2_ present in the blood. To compensate for this, the ongoing extension of this study, uses inhalation of a CO_2_ gas mixture. The different gender distributions between the cases and controls may also present a limitation. However, utilizing males only produces the same results, but the reduced number of cases yields lower statistical power. The lack of reliable tools to assess concussion severity prevents further separation of cases. The return to activity information could be a surrogate for severity, but the standard of care proved too heterogeneous. Often physicians would see a patient only for an initial assessment and then defer the return to activity decision to the athletic trainers, making a comparison between recovery rates as well as further data collection unreliable. Finally, since no imaging analysis was performed in this study, some subjects could have been suffering from undiagnosed structural injuries. Given the low severity of the reported injuries that appears unlikely, however, it is a possibility that would confound these results.

### Conclusion

Current concussion management standard of care relies on patient symptoms, neurocognitive evaluations, and physical performance testing (33). However, CBF alterations can persist after the clinical symptoms these evaluations are capturing have subsided (17, 34, 35). This suggests that the recovery is incomplete, and the patient may be at an increased risk for brain injury from further concussions, even when symptomatically recovered. This study echoes these previous reports, and highlights the need for an objective and quantitative physiological biomarker for the assessment of cerebral hemodynamic dysfunction following a concussion.

To our knowledge, this is the first study to capture multiple phases of hemodynamic dysfunction after a concussive injury using TCD. These results further highlight the complexity of concussive injury and support the belief of how vulnerable pediatric populations are to head injuries. The need for more quantitative methods to aid in the diagnosis of a concussion has been well established and these results are encouraging in the pursuit of that goal.

The pulsatile analysis is particularly important. The ability to capture dysfunction without the need for perturbation means that a quantitative biomarker of concussion can be captured without added stress on the patient. Over the course of treatment, the hypercapnia challenge can then be introduced to further track progress.

## Ethics Statement

This study was carried out in accordance with the recommendations of Declaration of Helsinki and The Western Institutional Review Board with written informed consent from all subjects. All subjects gave written informed consent in accordance with the Declaration of Helsinki. The protocol was approved by the Western Institutional Review Board (IRB #20141111).

## Author Contributions

CT had full access to all the data in the study and takes responsibility for the integrity of the data and the accuracy of the data analysis. Study concept and design: RH. Analysis: CT and ST. Interpretation of data: CT and RH. Drafting of the manuscript: CT. Critical revision of the manuscript for important intellectual content: RH, SW, and RD-A. Statistical analysis: CT. Technical, or Material Support: MO, NC, MR, IP, AS, JL, FS, and SW. Study Supervision: RH, JL, and CT.

## Conflict of Interest Statement

At the time that this research was conducted, CT, ST, MO, NC, MR, IP, AS, JL, SW, and RH were employees of, and either hold stock or stock options in, Neural Analytics, Inc. FC and RD-A have equity positions in Neural Analytics, Inc.
